# Axon terminal hypertrophy of striatal projection neurons with levodopa-induced dyskinesia priming

**DOI:** 10.3389/fnins.2023.1169336

**Published:** 2023-06-07

**Authors:** Takashi Nakamura, Haruo Nishijima, Fumiaki Mori, Iku Kinoshita, Tomoya Kon, Chieko Suzuki, Koichi Wakabayashi, Masahiko Tomiyama

**Affiliations:** ^1^Department of Neurology, Hirosaki University Graduate School of Medicine, Hirosaki, Japan; ^2^Department of Neuropathology, Hirosaki University Graduate School of Medicine, Hirosaki, Japan

**Keywords:** GABA, abnormal involuntary movement, synaptic plasticity, priming, levodopa-treated rats, synaptic vesicles, axon terminals

## Abstract

**Background:**

A rat model of levodopa-induced dyskinesia (LID) showed enlarged axon terminals of striatal direct pathway neurons in the internal segment of the globus pallidus (GPi) with excessive gamma-aminobutyric acid (GABA) storage in them. Massive GABA release to GPi upon levodopa administration determines the emergence of LID.

**Objectives:**

We examined whether LID and axon terminal hypertrophy gradually develop with repeated levodopa treatment in Parkinsonian rats to examine if the hypertrophy reflects dyskinesia priming.

**Methods:**

6-hydroxydopamine-lesioned hemiparkinsonian rats were randomly allocated to receive saline injections (placebo group, 14 days; *n* = 4), injections of 6 mg/kg levodopa methyl ester combined with 12.5 mg/kg benserazide (levodopa-treated groups, 3-day-treatment; *n* = 4, 7-day-treatment; *n* = 4, 14-day-treatment; *n* = 4), or injections of 6 mg/kg levodopa methyl ester with 12.5 mg/kg benserazide and 1 mg/kg 8-hydroxy-2-(di-n-propylamino)tetralin for 14 days (8-OH-DPAT-treated group; *n* = 4). We evaluated abnormal involuntary movement (AIM) scores and axon terminals in the GPi.

**Results:**

The AIM score increased with levodopa treatment, as did the hypertrophy of axon terminals in the GPi, showing an increased number of synaptic vesicles in hypertrophied terminals.

**Conclusion:**

Increased GABA storage in axon terminals of the direct pathway neurons represents the priming process of LID.

## 1. Introduction

Levodopa is the gold standard in treating Parkinson’s disease (PD) (Mercuri and Bernardi, 2005). However, with disease progression, patients experience troublesome levodopa-induced dyskinesia (LID) (Aquino and Fox, 2015). The onset of LID has been correlated with the over-reduction of the firing frequency and altered firing patterns in the internal segment of globus pallidus (GPi) in the primate model of PD (Papa et al., 1999; Boraud et al., 2001). Although the exact mechanism is unknown, it has been demonstrated that LID improves dramatically with GPi pallidotomy (Lang et al., 1997; Fine et al., 2000). These observations show that GPi may have a central role in LID.

We have previously demonstrated that intermittent levodopa administration to 6-hydroxydopamine (6-OHDA)-lesioned hemiparkinsonian rats causes LID-like abnormal involuntary movements (AIM) and axon terminal hypertrophy of striatal spiny projection neurons (SPNs) in the GPi (Nishijima et al., 2020). In this paper, to simplify the discussion of similarities between primates and rodents, we use the same terminology in rodents used in primates to discuss the globus pallidus subdivisions. We term the structure referred to as the entopeduncular nucleus in rodents as GPi according to widely accepted homology (Paxinos and Watson, 1998). We showed that the enlarged axon terminals in GPi of LID model rats originated from direct pathway neurons and contained high levels of gamma-aminobutyric acid (GABA) (Nishijima et al., 2020). Furthermore, excessive GABA was released into the GPi upon levodopa treatment, and the GABA release determined the emergence of LID (Nishijima et al., 2020). These results indicate that GABA storage in axon terminals of direct pathway SPNs and the resultant hypertrophy could explain the priming process of LID. However, such neuroplastic changes were found in an established LID model using a high dose of levodopa (50 mg/kg) in which even the first dose of levodopa-induced LID in all animals (Nishijima et al., 2020). This study explored whether the development of LID and the hypertrophy of axon terminals in the GPi gradually developed with repeated levodopa treatment and confirmed that GABA storage in axon terminals is associated with the priming process of LID.

## 2. Materials and methods

### 2.1. Animals

Male Wistar rats (Clea, Japan) weighing 280–320 g were used following the Hirosaki University School of Medicine’s Guidelines for Animal Experimentation issued and the Guide for the Care and Use of Laboratory Animals [National Institutes of Health (NIH), USA]. Animals were housed in a temperature-controlled room and exposed to 12 h light-dark cycles. The Hirosaki University School of Medicine reviewed and approved this animal study with the approval number: M20001. Foods were provided *ad libitum*. Every effort was made to minimize the number of animals and their suffering.

### 2.2. Unilateral 6-OHDA-lesion

Lesions in the dopaminergic system were generated on the right side of rats by 6-OHDA injection into the right medial forebrain bundle. The rat head was fixed in a stereotactic apparatus (David Kopf, USA) with the incisor bar set 3.3 mm below the horizontal after anesthesia with an intraperitoneal injection of pentobarbital (40 mg/kg body weight), medetomidine hydrochloride (0.02 mg/kg body weight) and midazolam (0.3 mg/kg body weight). Thirty minutes before the 6-OHDA injection, the rat was intraperitoneally injected with desipramine (25 mg/kg) to prevent the denervation of noradrenergic neurons. 6-OHDA was then injected through a stainless steel needle (0.4 mm outer diameter) that was inserted through a small burr hole on the right side of the skull. The needle tip was placed 4.5 mm posterior to the bregma, 1.2 mm lateral to the sagittal suture, and 8.5 mm ventral to the skull surface, according to the atlas of Paxinos and Watson (1998). 6-OHDA (8 μg/4 μl in saline with 0.01% ascorbic acid) was injected over 4 min, after which the needle was left in place for another 4 min to prevent backflow leakage.

Rats underwent rotational behavior testing after 2 weeks to evaluate the extent of 6-OHDA lesioning. Apomorphine (0.05 mg/kg) was administered subcutaneously, and rats were placed in a stainless-steel bowl 10 min later. After a 5-min accommodation period, the number of turns-to-the left (the side contralateral to the lesion) made by the rat was counted for 5 min. More than 20 contralateral turns indicated the loss of more than 99% of dopamine content in the striatum (Tanaka et al., 1999). We injected 6-OHDA in 35 rats. Twenty of the 35 injected rats passed the apomorphine test criteria and were included in this study.

### 2.3. Grouping and treatment

Five weeks post-operatively, 20 6-OHDA-lesioned rats were randomly allocated to receive saline injections (placebo group, 14 days; *n* = 4), injections of 6 mg/kg levodopa methyl ester combined with 12.5 mg/kg benserazide hydrochloride (levodopa-treated groups, 3-day-treatment; *n* = 4, 7-day-treatment; *n* = 4, 14-day-treatment; *n* = 4), or injections of 6 mg/kg levodopa methyl ester with 12.5 mg/kg benserazide and 1 mg/kg 8-hydroxy-2-(di-n-propyl amino)tetralin (8-OH-DPAT) for 14 days (8-OH-DPAT-treated group; *n* = 4). 8-OH-DPAT is a serotonin-1A receptor agonist, and its administration with levodopa could affect levodopa metabolism by suppressing dopamine release into the synaptic terminal (Kannari et al., 2001). We used 8-OH-DPAT because it has anti-LID effects in this rat model (Lindenbach et al., 2015). All groups received intraperitoneal injections twice daily for each dosing period, and all drugs [purchased from Sigma (Japan), except 8-OH-DPAT, obtained from Research Biochemicals International (USA)] were dissolved in saline. Based on previous reports, we set the therapeutic dose at 6 mg/kg levodopa, which can induce involuntary movements in all rats when a high degree of denervation is achieved (Lundblad et al., 2002; Nishijima et al., 2018).

### 2.4. Behavioral analysis

The behavioral effects of administering levodopa to 6-OHDA-lesioned rats were examined by recording AIM scores on the day of treatment 1, 3, 7, and 14, as Cenci and Lundblad (2007) reported. Briefly, rats were observed individually for 1 min every 20 min for 3 h. Two independent examiners blinded to the animal treatment conditions scored each rat on a severity scale from 0 to 4. Among the four AIM subtypes in 6-OHDA-lesioned levodopa-treated rats, axial dystonia, limb dyskinesia, and orolingual dyskinesia are reportedly equivalent to LID in patients with PD (Lundblad et al., 2002). In contrast, locomotor activity has been reported to be induced by both levodopa and long-acting dopamine agonists. Thus, it does not provide any specific measure of levodopa-induced motor complications (Lundblad et al., 2002). Thus, the total score of axial dystonia, limb dyskinesia, and orolingual dyskinesia (ALO AIM) was used as an index of LID severity.

### 2.5. Electron microscopy evaluation of synaptic areas in the GPi

**FIGURE 1:**
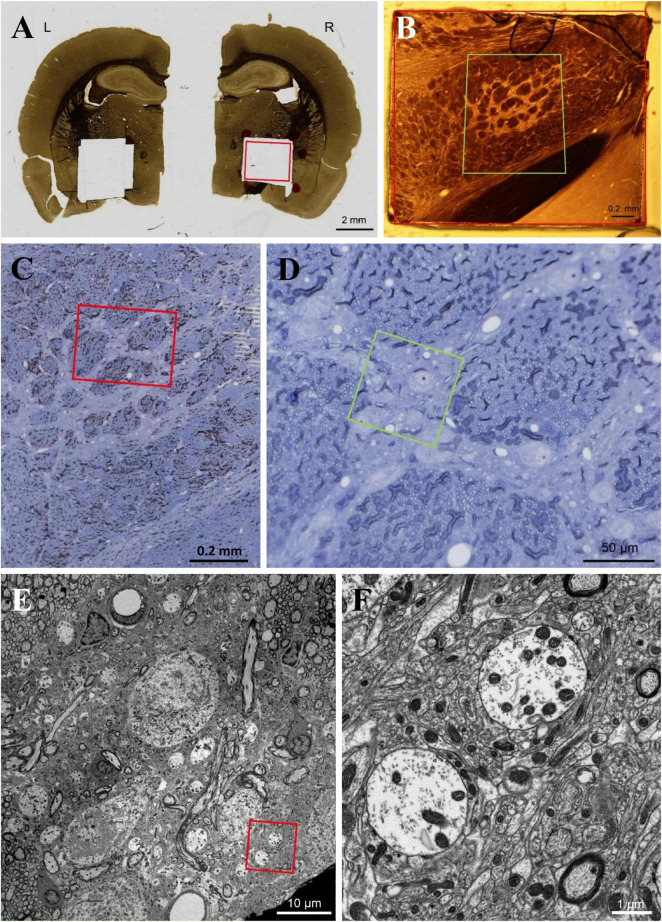
**(A–E)** We used flat-embedding sections between glass microscope slides that were pretreated with dimethyldichlorosilane (DMDCS), a silicone-based releasing agent (Aldes and Boone, 1984). The DMDCS prevents bonding of the resin to the glass slides so that after polymerization, the two slides are easily separated. Moreover, since the DMDCS coat is thin and transparent, routine light microscopic examination of each section for the GPi can be easily achieved and the plastic overlying the structures of interest can be used for subsequent semithin and ultrathin sectioning. This is a kind of old-fashioned method, however, a good method to identify the GPi. Scale bar, **(A)** 2 mm, **(B,C)** 0.2 mm, **(D)** 50 μm, **(E)** 10 μm, **(F)** 1 μm.

Twelve hours after the last injection, all rats (*n* = 20) were anesthetized with pentobarbital sodium (50 mg/kg) and transcardially perfused with 2.5% glutaraldehyde in 0.1 M phosphate buffer, pH 7.4. The brains were removed, and sections containing the GPi were cut at 50-μm-thickness with a vibratome. Sections were then treated with osmium tetroxide (1% in 0.1 M phosphate buffer), block-stained with uranyl acetate, dehydrated in graded series of ethanol, and flat-embedded on glass slides in Poly/Bed 812 Resin (Polysciences Inc., Warrington, PA, USA). Regions of interest containing the GPi were cut away (Figure 1A), glued on a flat-surfaced plastic block, and then cut into serial 0.5 μm-thick semithin sections (Figure 1B). Sections were stained with toluidine blue to identify the GPi (Figures 1C, D). After the GPi were identified, subsequent blocks were cut at 90–100 nm on an ultramicrotome (MT-7000, RMC Inc., Tucson, AZ, USA) and collected on copper grids (Figures 1E, F). Staining was performed on uranyl acetate drops, followed by lead citrate. Ultrastructural analyses were performed using a JEOL1230 electron microscope (JEOL Ltd., Tokyo, Japan). For quantitative evaluation, photomicrographs (x 15,000) were acquired from randomly selected electron microscope fields, and the area of the axon terminals making synaptic contacts with dendrites and the number of synaptic vesicles in the terminals was measured using NIH image software version 1.53 (National Institutes of Health, Bethesda, MD, USA). For each GPi, 11–17 pictures were taken randomly, and 2–4 of these pictures were selected for measurement where dendrites and nerve endings could be measured. The number of axon terminals on the dendrites, the area of the largest axon terminal in each image, and the number of vesicles in the terminal were recorded, and the average value for each rat was used for analysis. In this study, we evaluated nerve terminals forming symmetrical synapses, regarded as GABA endings.

### 2.6. Statistical analysis

Statistical analyses were performed using BellCurve for Excel version 3.21 (Social Survey Research Information Company, Ltd., Tokyo, Japan) and Excel (Microsoft Corporation, Redmond, WA, USA). The 3 h sum score of each AIM subtype and ALO AIM scores, the area and the number of axon terminals attached to dendrites in the GPi, and the number of vesicles were analyzed. Differences in AIM scores between groups were examined using the multi-way repeated measures ANOVA followed by the *post-hoc* Sheffe test. On analyses, levodopa-treated rats are integrated into one group, including all the rats in the particular treatment days (including 1–3 groups). Differences in the axon terminal area, density of axon terminals attached to a single dendrite, and number of synaptic vesicles in the intact and lesioned GPi side of all groups were examined by two-way repeated measures ANOVA with the *post-hoc* Tukey’s test. Spearman’s rank correlation coefficient examined regression analysis of area and synaptic vesicles. All data are expressed as mean ± standard error of the mean. *p* < 0.05 was considered statistically significant.

## 3. Results

### 3.1. AIM scores

**FIGURE 2:**
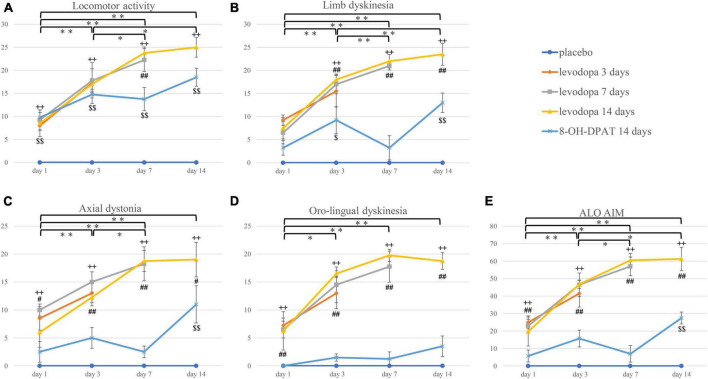
**(A–E)** The 3-h sum score of locomotor activity, axial dystonia, limb dyskinesia, orolingual dyskinesia, and ALO AIM score. *P < 0.05; ^**^P < 0.01 between treatment days in the integrated levodopa-treated group (including three groups). ^++^*P* < 0.01 between the integrated levodopa-treated group vs. the placebo group. ^#^*P* < 0.05; ^##^*P* < 0.01 between the integrated levodopa-treated group vs. 8-OH-DPAT group. ^$^*P* < 0.05; ^$$^*P* < 0.01 between the 8-OH-DPAT group vs. placebo group. ALO AIM, the total score of axial dystonia, limb dyskinesia, and orolingual dyskinesia; 8-OH-DPAT, 8-hydroxy-2-(di-n-propyl amino)tetralin.

The placebo group did not show any AIMs. In the levodopa-treated groups, there was an increase in all AIM scores with significant differences on days 3, 7, and 14 compared to day 1 (Figures 2A–E). Locomotor activity, limb dyskinesia, and ALO AIM also showed significant differences in the levodopa-treated groups between days 3 and 14 (Figures 2A, B, E). There were differences in the levodopa-treated group between days 3 and 7 except for oro-lingual dyskinesia (Figures 2A–C, E). There was also a significant difference between the levodopa-treated and placebo groups, the levodopa-treated and 8-OH-DPAT-treated groups in all the dyskinesia subtypes on treatment days 3, 7, and 14 (Figures 2B–E). Locomotor activity, axial dystonia, limb dyskinesia, and ALO AIM showed a difference between the 8-OH-DPAT and placebo group on days 14 (Figures 2A–C, E).

### 3.2. Axon terminal area surrounding dendrites of GPi neurons and number of synaptic vesicles in the axon terminals

**FIGURE 3:**
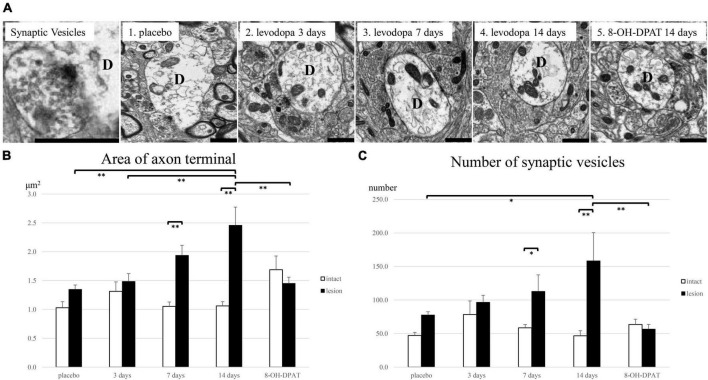
Electron microscopic evaluation in the internal segment of globus pallidus (GPi) in unilaterally 6-hydroxydopamine(OHDA)-lesioned rats treated with saline for 14 days (placebo group; *n* = 4), levodopa (levodopa-treated group, 3-day; *n* = 4, 7-day; *n* = 4, 14-day; *n* = 4) or levodopa and 8-hydroxy-2-(di-n-propyl amino) tetralin for 14 days (8-OH-DPAT-treated group; *n* = 4). **(A)** Repeated levodopa treatment induced enlargement of axon terminals surrounding dendrites of GPi neurons on the lesioned side in 6-OHDA-lesioned hemiparkinsonian rats. Synaptic vesicles are found within axon terminals attached to dendrites. The 8-OH-DPAT-treated group suppressed the hypertrophy induced by levodopa. **(B)** The largest axon terminal area surrounding the dendrites of GPi neurons on the lesioned side significantly enlarged compared with the intact side in the levodopa-treated groups with longer treatment (7- and 14-day). **(C)** The number of synaptic vesicles in the lesioned side increased significantly compared with the intact side in the levodopa-treated groups with longer treatment (7- and 14-day). Asterisks indicate statistically significant differences (*P < 0.05; ^**^P < 0.01). Error bars represent standard errors of the mean. Scale bar, 1 μm. D, dendrite.

The area of the axon terminal surrounding the dendrites of GPi neurons on the lesioned side enlarged in the 7- and 14-day levodopa-treated group compared with the intact side. The 3-day levodopa-treated and 8-OH-DPAT-treated groups showed no increase in axon terminal areas on the lesioned side compared to those on the intact side (Figures 3A, B). On the lesioned side, the 14-day levodopa-treated group showed a significant increase in the axon terminal area compared to the placebo and 3-day levodopa-treated and 8-OH-DPAT-treated groups (Figure 3B). The number of synaptic vesicles on the lesioned side was also higher than those on the intact side in the 7- and 14-day levodopa-treated groups. The 3-day levodopa-treated and 8-OH-DPAT-treated groups showed no significant increase in vesicle numbers compared to the placebo group. The 14-day levodopa-treated group showed a significant increase in synaptic vesicles compared to the placebo and 8-OH-DPAT-treated groups (Figure 3C) on the lesioned side. 1–6 axon terminals were attached to dendrites in each GPi. There are no differences between experimental groups in the density of the boutons attached to a dendrite. The levodopa administration period did not impact the density of the boutons. Regression analysis of the area and synaptic vesicles showed a positive correlation.

## 4. Discussion

This study showed that repetitive levodopa treatment in Parkinsonian rats induced LID and hypertrophy of the axon terminals in the GPi with treatment duration dependence; the more extended dosing period brought the larger terminals. In addition, the number of synaptic vesicles in the terminals also increased. Furthermore, 8-OH-DPAT suppressed the enlargement of the axon terminals and the increase in synaptic vesicles. Thus, the observation that progressive LID and concomitant gradual progressive hypertrophy of axon terminals in GPi with increased synaptic vesicles support our hypothesis that GABA storage in the axon terminals of direct pathway neurons plays a role in the priming process of LID (Nishijima et al., 2020).

Dopamine acts on D1 and D2 dopamine receptors in the striatum, mainly expressed in the striatum’s direct and indirect projection neurons, respectively (Albin et al., 1989). The concept that dyskinesia is caused by repetitive abnormal stimulation of D1 receptors has been previously reported (Obeso et al., 2000; Bezard et al., 2001). In the dopamine-dominated striatum, other studies showed persistent D1 receptor supersensitivity (Corvol et al., 2004) and increased D1 receptor-mediated signaling (Gerfen et al., 2002). Furthermore, D1 receptor hypersensitivity was associated with altered synaptic plasticity at corticostriatal synapses in the LID model rodents (Picconi et al., 2003). Chronic administration of levodopa has been reported to induce abnormalities in GABAergic transmission from the direct pathway (Gerfen et al., 1990), as evidenced by increased expression of dynorphin with GABA and increased glutamate decarboxylase 67 (an isoform of the enzyme that synthesizes GABA) mRNA (Nielsen and Soghomonian, 2004). Thus, hypertrophy of axon terminals of direct pathway striatal neurons and accumulation of GABA in levodopa-treated rats were thought to be strongly associated with LID expression.

We previously showed that repeated high-dose levodopa (50 mg/kg) administration to 6-OHDA-lesioned rats resulted in the enlargement of axon terminals surrounding the dendrites of GPi neurons (Nishijima et al., 2020). Those axon terminals were vesicular GABA transporter-positive and dynorphin- and substance-P-positive, indicating that they belong to the direct pathway striatal SPNs (Nishijima et al., 2020). In addition, the terminals were filled with numerous synaptic vesicles (Nishijima et al., 2020). Accordingly, we showed that the abnormal GABA storage in the terminals of the neurons of the direct pathway might reflect the LID priming. However, the study had a limitation. We administrated levodopa at a very high dose, and therefore, even the first dose of levodopa-induced strong dyskinetic movements in all animals. Thus, we could not demonstrate the direct relationship between progressive dyskinetic movements and the development of hypertrophy of the axon terminals. In the same study, we showed massive GABA release to the GPi upon levodopa dosing in LID model rats (Nishijima et al., 2020). Therefore, the excessive GABA release likely accounted for the emergence of LID. Here, we added new evidence that exacerbation of dyskinesia and progressive enlargement of the axon terminals coincide using a therapeutic levodopa dose (6 mg/kg). However, this study did not investigate the origin of the hypertrophied axon terminals.

Our results show that LID may be controlled by inhibiting the excessive release of GABA. Basic research has shown that GABA_B_ agonist administration improved motor function in the rat model of 1-methyl-4-phenyl-1,2,3,6-tetrahydropyridine-induced PD (Tyagi et al., 2015). Other studies showed that dopaminergic denervation had induced a loss of negative feedback via the GABA_B_ receptor in the axon terminal of direct pathway striatal spiny projection neurons, leading to hyperactivity of the neurons and the development of LID (Borgkvist et al., 2018). Thus, GABAergic modulation may be the promising target for treating LID.

This study has other limitations: we examined the GPi pathology associated with LID in a rodent PD model with neurotoxin-induced hemi-dopaminergic denervation. Moreover, we confirmed an increased accumulation of synaptic vesicles in GPi, but not their functional release with LID. Further studies in primate and genetic models measuring GABA release in GPi are warranted to confirm our concept that direct pathway axon terminal hypertrophy in the GPi is the pathological hallmark of LID.

In conclusion, we revealed that the therapeutic levodopa treatment contributed to axon terminal pathological changes in the GPi dependent on treatment duration. This process may reflect LID priming.

## Data availability statement

The raw data supporting the conclusions of this article will be made available by the authors, without undue reservation.

## Ethics statement

This animal study was reviewed and approved by the Animal Research Committee of Hirosaki University.

## Author contributions

TN, HN, and MT conceived the presented idea. TN, HN, and FM experimented. TN wrote the manuscript with support from TK, IK, CS, and KW. All authors discussed the results and contributed to the final manuscript.
